# Future soil moisture and temperature extremes imply expanding suitability for rainfed agriculture in temperate drylands

**DOI:** 10.1038/s41598-017-13165-x

**Published:** 2017-10-10

**Authors:** John B. Bradford, Daniel R. Schlaepfer, William K. Lauenroth, Charles B. Yackulic, Michael Duniway, Sonia Hall, Gensuo Jia, Khishigbayar Jamiyansharav, Seth M. Munson, Scott D. Wilson, Britta Tietjen

**Affiliations:** 1 U.S. Geological Survey, Southwest Biological Science Center, Flagstaff, AZ 86001 USA; 2 University of Basel, Section of Conservation Biology, 4056 Basel, Switzerland; 3 School of Forestry and Environmental Studies, Yale University, New Haven, CT 06511 USA; 4 U.S. Geological Survey, Southwest Biological Science Center, Moab, UT 86001 USA; 5 Center for Sustaining Agriculture and Natural Resources, Washington State University, Wenatchee, Washington, 98801 USA; 6 SAH Ecologia LLC, Wenatchee, Washington, 98801 USA; 7 Chinese Academy of Sciences, Institute of Atmospheric Physics, Beijing, 100029 China; 8 Department of Forest, Rangeland and Watershed Stewardship, Colorado State University, Fort Collins, CO USA; 9 Department of Biology, University of Regina, Regina, Saskatchewan S4S 0A2 Canada; 10 Freie Universität Berlin, Biodiversity and Ecological Modeling, 14195 Berlin, Germany; 11 Berlin-Brandenburg Institute of Advanced Biodiversity Research (BBIB), 14195 Berlin, Germany

**Keywords:** Projection and prediction, Environmental impact

## Abstract

The distribution of rainfed agriculture, which accounts for approximately ¾ of global croplands, is expected to respond to climate change and human population growth and these responses may be especially pronounced in water limited areas. Because the environmental conditions that support rainfed agriculture are determined by climate, weather, and soil conditions that affect overall and transient water availability, predicting this response has proven difficult, especially in temperate regions that support much of the world’s agriculture. Here, we show that suitability to support rainfed agriculture in temperate dryland climates can be effectively represented by just two daily environmental variables: moist soils with warm conditions increase suitability while extreme high temperatures decrease suitability. 21^st^ century projections based on daily ecohydrological modeling of downscaled climate forecasts indicate overall increases in the area suitable for rainfed agriculture in temperate dryland regions, especially at high latitudes. The regional exception to this trend was Europe, where suitability in temperate dryland portions will decline substantially. These results clarify how rising temperatures interact with other key drivers of moisture availability to determine the sustainability of rainfed agriculture and help policymakers, resource managers, and the agriculture industry anticipate shifts in areas suitable for rainfed cultivation.

## Introduction

Meeting the nutritional demands of a burgeoning human population in coming decades will require shifts in the distribution of global cultivation^1^. Approximately 1.5–1.6 billion hectares, or roughly 12–15% of the earth’s terrestrial land surface, are currently in cultivation or exist within a matrix of substantial cultivation, and in many regions cultivation is nearly ubiquitous^2,3^. Expansion of cultivation into newly suitable locations is an anticipated societal response to both changing climate^4^ and shifting geographic patterns of human populations^5^. Strategies for adapting our agricultural systems to climate change^6^ include enhanced irrigation^7–9^, although future water supplies for irrigation are uncertain, especially as societal water demand grows and changing climatic conditions alter both temperature and precipitation patterns^10^. Consequently, rainfed agriculture, which currently represents approximately 75% of global croplands^11^, is likely to remain a major component of the global food system. As cultivated lands shift^4^, the importance of rainfed agriculture will become especially pronounced for dryland areas where irrigation infrastructure and water supply are already limited. Quantifying the climatic and edaphic controls of rainfed cultivation in drylands is necessary to facilitate accurate forecasts of local, national, and global food supply^12^. In addition, understanding the potential future distribution of agricultural land use is important to forecast the impact of human activities on a range of ecological processes and services^13^. Cultivation is a transformative land-use practice that influences ecosystem water balance^14^, carbon dynamics^15^, and nutrient cycling^16^, and modifies landscape attributes such as biodiversity, habitat connectivity, and potential migration pathways^17^.

The suitability of drylands for rainfed agriculture is dictated by interactions among temperature, precipitation, and soil texture^12,18–20^ that are not well estimated by mean climate conditions alone^21–23^. Climatic variables that influence the distribution of rainfed agriculture include the degree of overlap between warm and wet seasons^24^, growing degree days, aridity, and winter temperatures^19,25^. Studies of crop yields have indicated that high temperature extremes, which lead to high vapor pressure deficits, can decrease rainfed crop yields in a variety of crops and regions^4,26–32^, especially during sensitive periods of crop development^33^. In addition, the adverse impacts on crop yields appear to be mediated by transient deficits of soil water availability, rather than directly by temperature^12,21^. Despite this evidence for interactive effects of temperature extremes and transient soil moisture on crop yields, the roles that these variables play in dictating overall areas suitable for agriculture have not been explored. Understanding these effects is particularly important considering expectations for increasing temperature stress^31,32,34^ throughout the 21^st^ century.

Climate change is expected to decrease agricultural suitability at the global scale, although many studies have suggested that the most negative outcomes are likely to occur in tropical and sub-tropical systems^7,32,33,35,36^. By contrast, climate change impacts on agricultural suitability are uncertain for temperate regions^37^ that support a majority of global agricultural land^38^ and tend to have higher yields than tropical areas due in large part to higher soil fertility^39^. Globally, the temperate zone accounts for roughly 1/6 of cultivation and represents approximately 31% and 17%, of cultivated areas for wheat and maize, respectively^40^. Understanding the controls over agricultural suitability and the potential impact of climate change on the distribution of suitable areas is especially challenging in temperate drylands because suitability in areas with high annual temperature amplitude is driven by complex interactions among seasonal and soil-depth patterns of both soil moisture and temperature. Plant growth and overall agricultural suitability in these areas is restricted to the mostly frost-free, warm season, which may not coincide with the period of highest soil moisture availability.

Here, we examined climate and soil texture controls of rainfed agriculture suitability in temperate drylands. Our goal was to assess how climate change, and particularly changing weather extremes, may impact the distribution and abundance of areas suitable to support rainfed agriculture in temperate dryland regions. First, we examined current climate, weather, and edaphic controls of rainfed agriculture suitability in temperate drylands, and we identified the metrics of daily soil moisture availability and weather extremes that were most strongly related to the current distribution of rainfed agriculture, from a list of potential explanatory variables used by previous studies (SI Appendix, Table S1). Because local demand for food influences the distribution and abundance of cultivation^5^, we also included a static metric of access to markets in our candidate variables. Second, we quantified how potential climate change-driven shifts in both weather extremes and transient soil moisture conditions may alter the abundance and extent of areas in temperate drylands that can support rainfed agriculture.

## Results

### Climate and soil moisture controls over rainfed agriculture

**Figure 1:**
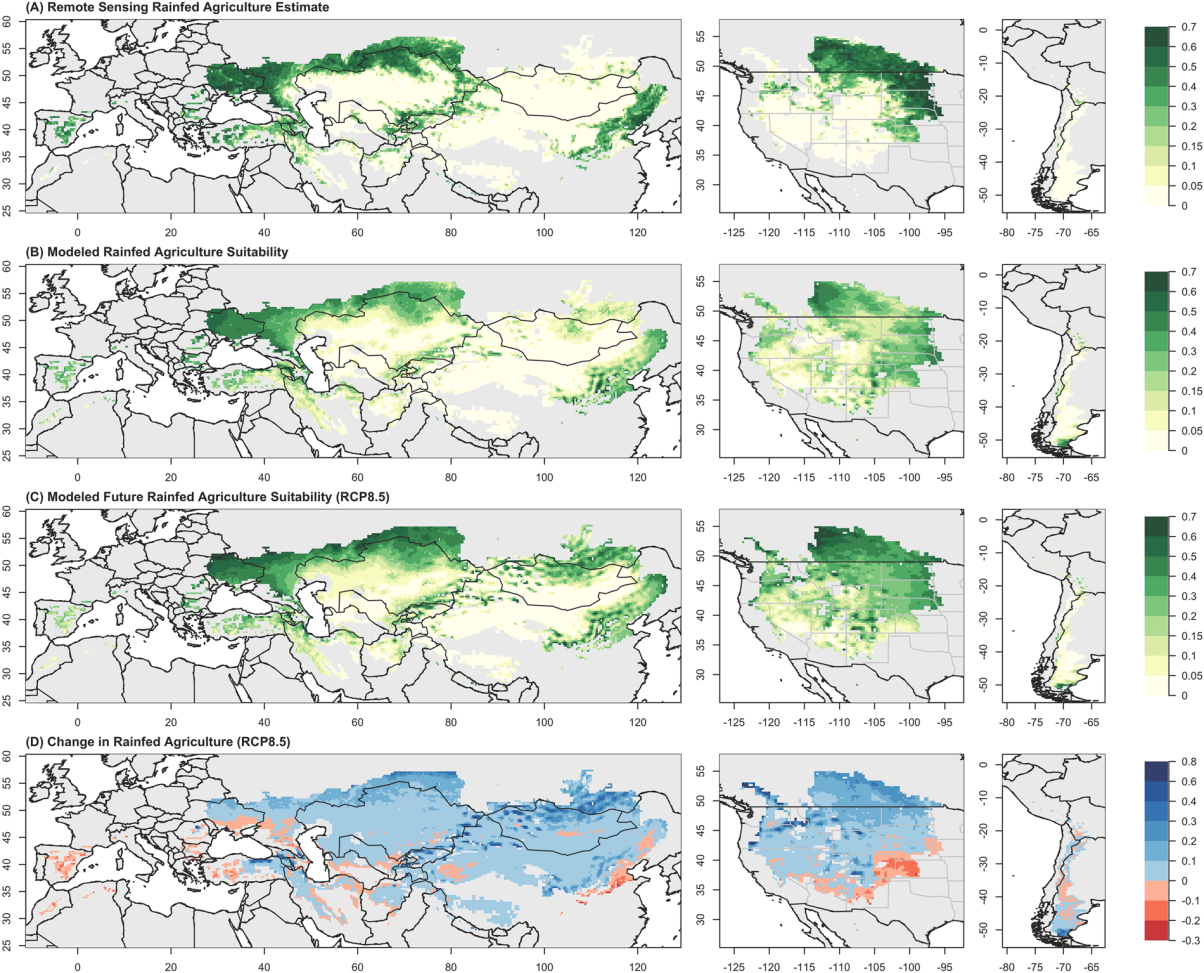
Rainfed agriculture (expressed as proportion of land area) in temperate drylands estimated from: (**A**) remote sensing (e.g. realized abundance), (**B)** predicted under current conditions from a statistical model (e.g. potential abundance), (**C**) predicted under future conditions (median GCM, RCP8.5), and (**D**) change in prevalence between future and current climate. Created in R version 3.3.2. (www.R-project.org/).

We examined how remotely-sensed measures of current rainfed agriculture in temperate drylands (Fig. 1A) related to seven candidate driving variables representing climate, weather extremes, soil moisture and access to markets as a measure of demand for food (SI Appendix, Table S1). Comparison of 10 *a priori* candidate models (SI Appendix, Table S2) built upon variables identified by independent regional optimization (see Methods) identified a best model utilizing three standardized variables and two interactions (SI Appendix, Table S2) that yielded unbiased estimates of rainfed agriculture (r^2^ = 0.55; SI Appendix, Figure S1) under current climate conditions:

$$\begin{array}{c}{\boldsymbol{RFA}}=logit(-2.4+1.2{\boldsymbol{WDD}}-0.12{\boldsymbol{TMAX}}+0.68{\boldsymbol{MKT}}\\ \quad \quad \quad \,\,-0.26{\boldsymbol{WDD}}\,\ast \,{\boldsymbol{MKT}}-0.07{\boldsymbol{WDD}}\,\ast \,{\boldsymbol{TMAX}})\end{array}$$

where RFA is proportion rainfed agriculture within a 0.3125-degree raster cell, WDD is wet degree days (degree days when shallow soils are wet; SI Appendix, Table S1), TMAX is the frequency of extreme hot days (maximum temperature >34 **°**C), and MKT is market access.

**Figure 2:**
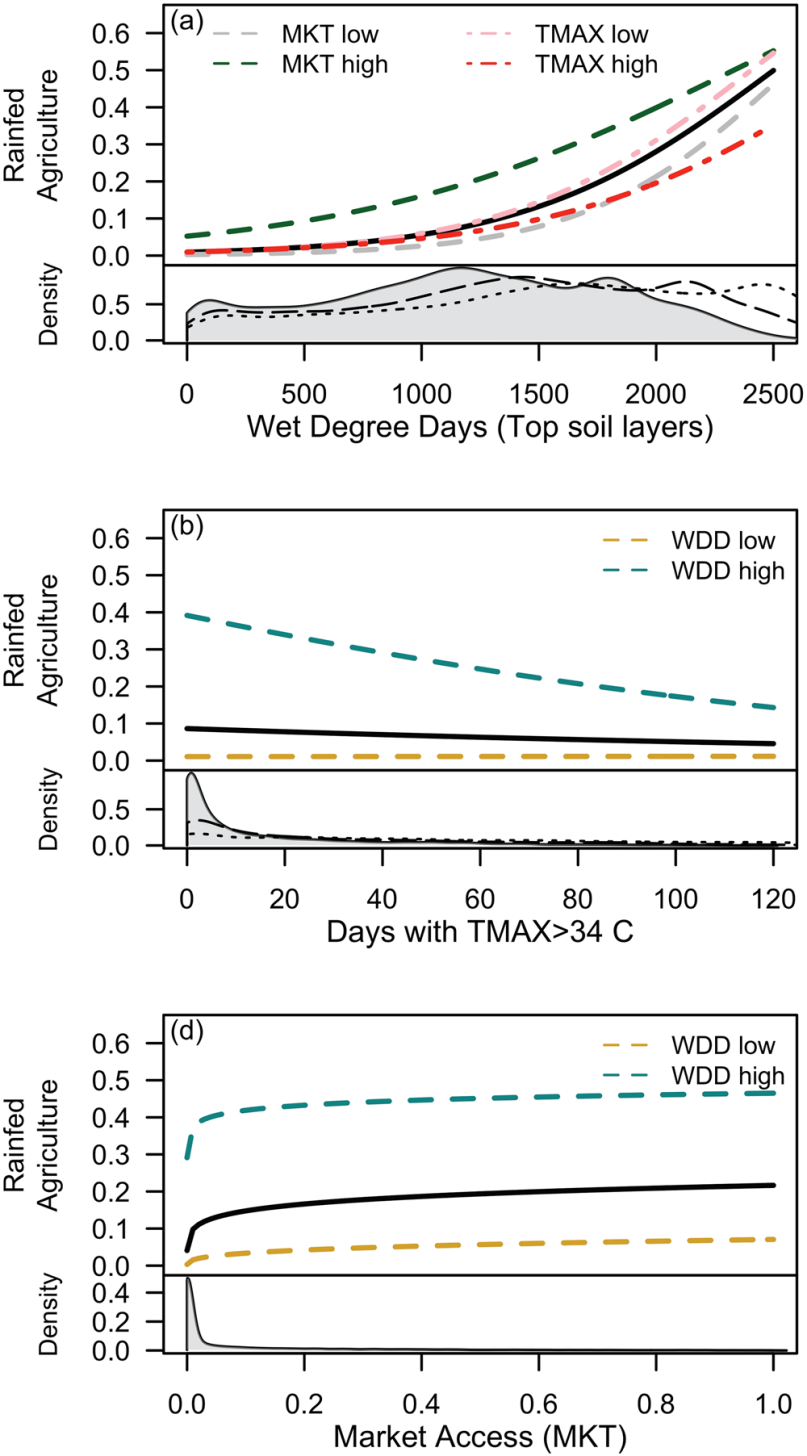
Controls over rainfed agriculture, represented by relationships between rainfed agricultural abundance and (**a**) wet degree days (WDD; growing degree days when soil water potential >-1.5 MPa in the top 20 cm of soil); (**b**) days with maximum temperature (TMAX) greater than 34 **°**C; and (**c**) access to markets (MKT). Black lines are mean response and colored dashed or dotted lines illustrate interactions by showing response under high or low (90th or 10th percentiles, respectively) of other driving variables. Gray areas provide insight into how climate change may alter these important driving variables, by depicting the distribution of temperate drylands relative to each driving variable, WDD in (**a**), TMAX in (**b**), MKT in (**c**), and lines depict forecasted future distributions (medians GCM forecast from RCP 4.5 and RCP8.5).

Our best model contained just two variables representing soil moisture and climate, suggesting that soil and climatic controls over agricultural suitability can be effectively represented by the interactive effects of the long-term mean annual frequency of wet degree days in the top soil layers and the long-term mean annual number of days with maximum temperature greater than 34 °C. Rainfed agriculture was positively related to wet degree days and market access, and negatively related to extreme hot days (Fig. 2). The positive relationship with wet degree days was the most powerful predictor of rainfed agriculture and non-linear such that rainfed agriculture is most responsive in locations where wet degree days are high (e.g. above ~1200 °C × days). In addition, the influence of wet degree days interacted with extreme hot days and market access (Fig. 2a). Specifically, in locations where wet degree days are low (e.g. <1000 °C × days), rainfed agriculture is also low regardless of extreme hot days or market access. Rainfed agriculture was negatively related to extreme heat (Fig. 2b), and that relationship is strongly influenced by the interaction with wet degree days. Specifically, rainfed agriculture is very low in cells exposed to a high frequency of extreme hot days regardless of wet degree days, whereas rainfed agriculture in areas with a low frequency of extreme hot days is strongly, and positively, related to wet degree days. Market access is positively associated with increased rainfed agriculture (Fig. 2c), although the response of rainfed agriculture to market access is most pronounced at very low (e.g. <0.05) market access (which are common in the data; Figure S2). By contrast, variation in market access from 0.05 to 1 have relatively modest impacts on the abundance of rainfed agriculture. Furthermore, the difference in rainfed agriculture abundance between high and low market access was greatest at intermediate levels of wet degree days (Fig. 2a). Within the full set of 10 candidate models (SI Appendix, Table S2), coefficients for wet degree days (1.15–1.33; best model, 1.18) and for markets access (0.68–0.87; best model, 0.68) were all positive and varied little, while 4 out of the 6 models that included coefficients for temperature extremes estimated them as negative (−0.26 to +0.37; best model, −0.12), underscoring the strong relationships between these independent variables (WDD and MKT) and rainfed agriculture.

**Figure 3:**
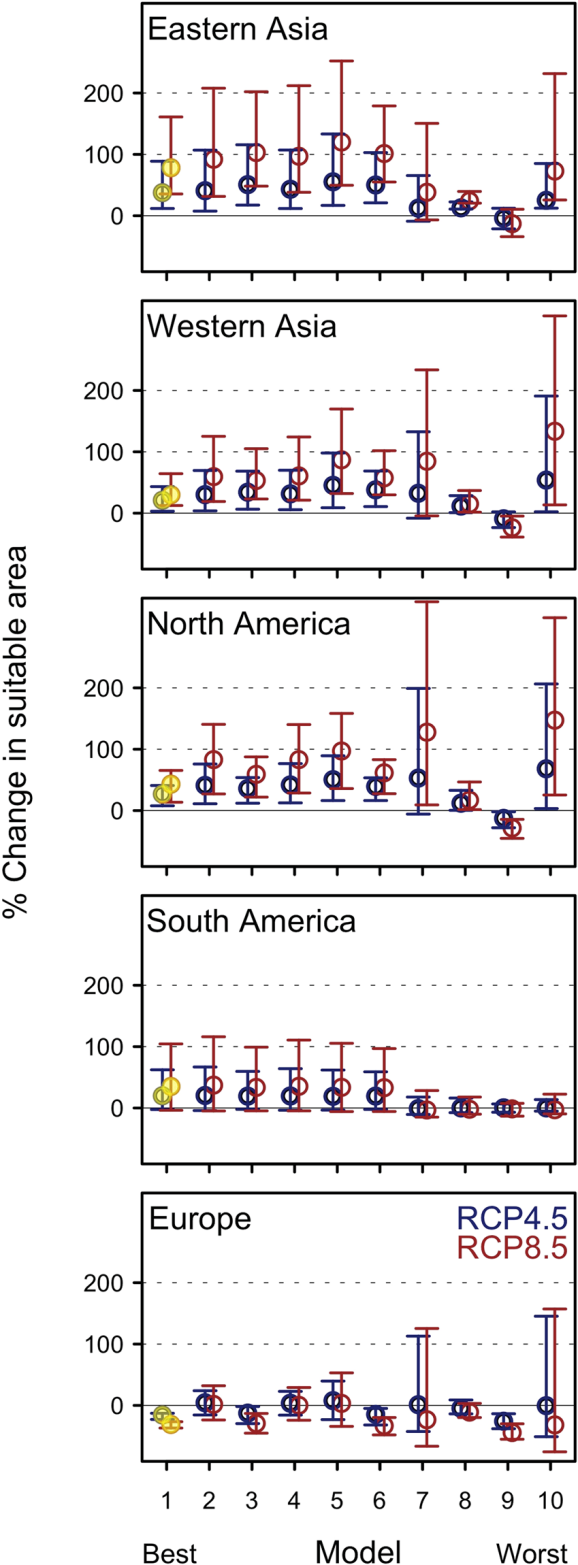
Estimated regional percent change in land area suitable for rainfed agriculture under future climatic conditions for all 10 statistical models examined (Table S2), with models ranked from best performing (#1, median changes highlighted in yellow) to worse performing (#10.) Projections are shown by region for RCP 4.5 (blue) and RCP 8.5 (red). Circles are change estimates for the median GCM and lines show range between rank 2 and rank 15 (out of 16 GCMs examined) for each RCP.

Our overall estimates of land area suitable for rainfed agriculture is moderately influenced by the assumptions about the cultivated areas within pixels classified as agriculture (see Methods). We estimate approximately 2.32 million km^2^ suitable for rainfed agriculture across all temperate dryland regions, close to the 2.51 million km^2^ of realized rainfed agriculture estimated by remote sensing^2^. Our estimates (Fig. 3 black bar) were approximately 9%, 14%, and 19% less than remotely-sensed estimates in North America, West Asia and Europe, respectively, 7% more in East Asia, and dramatically more (~600%) in South America. The large difference in South America indicates that our climatic and edaphic drivers suggest a potential for more widespread rainfed agriculture in the temperate dryland portions of South America^19^.

Predictions based on wet degree days, extreme temperatures and market access (SI Appendix, Figure S2) successfully reproduced current geographic patterns of rainfed agriculture estimated from remote sensing (RMSE = 13.7%; SI Appendix, Figure S1a). In general, the spatial gradients in predicted agricultural suitability are very similar to observed gradients of agriculture abundance (Fig. 1, SI Appendix, Figures S1 and S3). Areas with the highest density in East Asia are focused on the central part of northeastern China with small pockets of rainfed agriculture in the western part of the region. In western Asia, rainfed cultivation is most abundant in a broad east-west band extending from north of the Black Sea east across southern Russia, with smaller areas to the south from Kazakhstan to Turkey. Rainfed agriculture is most abundant in North America throughout the northern Great Plains, from the central United States into the southern parts of Manitoba, Saskatchewan, and Alberta in Canada. Predictions for each major region based on the model fit without that region assess the model’s capability to accurately predict into novel locations and climates, and those predictions are also good (RMSE = 15.5%), although they display a slight bias toward under-estimation of rainfed agriculture in areas of high agricultural abundance (SI Appendix, Figure S1b). Likewise, while our statistical model correctly identified the locations within Asia and North America that are highly suitable for rainfed agriculture, the actual intensity of rainfed agriculture within those regions was, in some places, higher than modeled (SI Appendix, Figure S3). These discrepancies underscore that decisions about rainfed cultivation are influenced by factors not represented in this analysis such as economic conditions other than market access or incentives driven by governmental subsidies for agriculture, competing alternative land uses, irrigation water cost and availability, and terrain or soil limitations not accounted for here. Despite these modest differences between estimated suitable area and estimated actual rainfed agriculture, the extremely low bias displayed by the relatively simple model identified here suggests that it provides a useful framework for examining the influence of climate variables and the potential consequences of changing climatic conditions for rainfed agricultural suitability.

### Climate change impacts on the distribution of areas suitable for rainfed agriculture

Contrasting current and future estimates of total area suitable for rainfed agriculture (included both cultivated and uncultivated) suggests potential increases for most regions. Compared to current modeled suitable area, our best model predicted that climate change represented by the median GCM under RCP8.5 at the end of the 21^st^ century (2070–2099) would result in a 41% increase in global temperate dryland area suitable to support rainfed agriculture (~0.96 million km^2^ based on current sub-pixel abundance assumptions, SI Appendix, Figure S4) (18% increase under RCP4.5; Fig. 3), assuming current market access. Under RCP8.5, we forecast suitable area to increase in East Asia by 78% (0.34 million km^2^), in West Asia by 30% (0.35 million km^2^), in North America by 43% (0.26 million km^2^), in South America by 35% (0.02 million km^2^), and decrease in Europe by 31% (0.014 million km^2^). The directions of these predictions for change in suitability were generally robust to uncertainty in both statistical model structure and future climate represented by variation among two emissions scenarios and 16 climate models (Fig. 3 and Figure S8), although variability in projected change in suitable area was greater in some regions (e.g. Eastern Asia and South America) than others. Uncertainty in statistical model structure had less influence than uncertainty in climate on predictions of rainfed agriculture suitability at the global scale and regional scale for all regions except North America (SI Appendix, Table S3). The top several models all projected increased suitability in Eastern and Western Asia and North and South America, while projections for Europe ranged between no change and ~40% decline. In all regions, estimates from the best model fell in the middle of the suite of projections from all models, indicating that the best model does not generate extreme predictions. Regional estimates of change in land area suitable to support rainfed agriculture (Fig. 3) increased under both RCPs and the low, middle, and high GCM ranks in East Asia (RCP4.5 range 12% to 89%; RCP8.5 range 35% to 161%), West Asia (RCP 4.5 range 3% to 43%; RCP 8.5 range 12% to 64%), and North America (RCP4.5 range 8% to 41%; RCP8.5 range 14% to 65%). Within these regions with overall increases, substantial decreases in suitability for rainfed agriculture are projected in many smaller areas, notably the southern great plains of North America and the southeastern portion of East Asia (Fig. 1). Anticipated declines in suitable area in Europe were also robust across RCPs and GCMs, ranging from −19% to −10% under RCP4.5 and −30% to −22% under RCP8.5 (Fig. 3). In South America, estimates of percent change ranged from −3% to 62% for RCP4.5 and −4% to 104% for RCP8.5.

## Discussion

Our results identify two important environmental influences over rainfed agriculture in temperate dryland areas. First, climate variability and weather events, expressed here as long-term average annual number of days with extreme heat, exert important control over suitability for rainfed agriculture, a process that may not be well represented by studies focusing only on mean climatic conditions or using a monthly time scale analysis. While extreme heat has a recognized impact on agricultural yields^4,26–30^, its role in restricting the distribution of areas suitable for rainfed agriculture has not been previously demonstrated. Second, extreme heat interacts with the dominant control exerted by transient soil moisture availability in these dryland regions such that rainfed agriculture can be restricted by any combination of dry conditions or extreme heat. These interacting influences are especially important in the context of climate change, because a growing potential for extreme heat events, more frequent ecological drought periods, and enhanced aridity in drylands are among the most reliable aspects of climate projections^41^ (Figure S8).

The long-term average annual sum of wet degree days in the top soil layers was the most influential variable in our best model; rainfed agriculture is non-existent where wet degree days are very low, but reliably more abundant where wet degree days are high. The importance of wet degree days is consistent with previous studies that have related cropland to growing degree days and overall aridity^19,25^, and studies showing adverse drought impacts on crop yields^33^. Wet degree days integrate important aspects of both temperature and moisture limitations, and was identified in our statistical model selection as a more effective predictor of rainfed agriculture than variables relating directly to either growing degree days or precipitation. Historical trends in climate extremes over the past few decades have adversely impacted agricultural production, although those impacts are masked by advances in agronomic technology, such as genetic crop improvements^12,29^. However, crop improvements that have increased rainfed crop yields in recent decades may be associated with greater drought vulnerability^33^.

We found that the number of days with maximum temperature greater than 34**°**C provided a useful metric for identifying areas with adequate wet degree days that are not suitable for agriculture because of frequent extreme high temperatures. Abundant evidence implies that crop yields are negatively impacted by extreme high temperatures^22,27,28,34,37^. However, our results are the first to illustrate that high temperature extremes provide a geographic limitation to rainfed agriculture, and in particular that the occurrence of episodic extreme heat events appears to restrict cultivation even in relatively cool temperate regions. Although we used 34 °C as our metric of potential heat stress, a value consistent with previous work^34^, we also examined a higher 40 °C threshold and found similar relationships with current rainfed agriculture abundance and similar impacts on future suitability (results not shown). As temperatures continue to rise in coming decades, this limitation will have potentially important negative consequences for future global food supply^42,43^.

Consistent with our hypothesis about the positive influence of proximity to population centers, our model selection indicated a positive relationship with market access. Although market access patterns may shift in the future, we did not attempt to anticipate those changes and, for the purpose of this analysis, assumed that the geographic patterns of market access will not change in the future. The inclusion of market access in our best model suggests that it provides useful information that can complement climate and ecohydrological metrics to enhance future studies of agricultural suitability.

For temperate dryland regions, the importance of both wet degree days and high temperature extremes, and the interaction between them, suggests a simple framework for interpreting both historical relationships between climate and agriculture, as well as for developing future projections of climate change impacts that explicitly incorporate edaphic influences over moisture availability^23^. For example, recent climatic warming trends have been related to decreased crop yield in many temperate regions, including soy and maize in North America and Asia, and wheat in Asia, and increased yield in other areas, including wheat in North America and rice in the Northern parts of Asia^6^. While most of these yield patterns were consistently linked to temperature trends, growing season precipitation trends, which are less consistent than temperature trends, showed weak relationships with crop yield at global scales^29^ but are likely much more important in water-limited arid and semiarid climates.

Climate change appears likely to increase suitability for rainfed agriculture at high latitude, and to a lesser extent high elevation, locations (Fig. 1 and SI Appendix, Figure S5), consistent with previous work^19,25,44^. In particular, the northern portions of East Asia, West Asia and North America and the southern portion of South America all display increases in suitability for rainfed agriculture. Some portions of these areas of increasing suitability are already heavily cultivated, while others have relatively low current prevalence of cultivated agriculture. This increased suitability is driven primarily by increases in temperature that when combined with slight precipitation increases, result in higher wet degree days in the top soil layers (SI Appendix, Figure S6). However, climate models also forecast substantial increases in the occurrence of extreme high temperatures for these regions, (SI Appendix, Figure S7), which limits the potential increases in suitability for rainfed agriculture and contributes to the predicted suitability decreases in some locations. Although we did not explicitly restrict our assessment of suitability by topography, areas of extreme topographic relief (defined here as >2000 m elevation range within one of our cells) represented only approximately 1% of total suitable areas under either current or future conditions, suggesting that our estimates of newly suitable future areas do not contain substantial amounts of mountainous areas with extreme topography.

Despite our overall result of increased area suitable for rainfed agriculture in most temperate regions, our analysis also indicated locations within each region that are currently heavily cultivated, but will become less suitable in the future. Most of the temperate dryland cultivated areas in Europe are expected to experience declining suitability. Similar declines are predicted for southern China, many southern portions of West Asia around the Black Sea and in southern Kazakhstan, and the southeastern U.S. Great Plains, especially the northern parts of the shortgrass steppe in eastern Colorado and western Kansas. Previous analysis suggested that rainfed agriculture was restricted to conditions where AET/PET > 0.5^25^, but we found that dryland agriculture can occur in drier conditions. Our results suggest that ~1.1 million km^2^ of rainfed agriculture exists in areas with AET/PET < 0.5, and 0.66 million km^2^ where AET/PET < 0.4. The feasibility of rainfed agriculture in these semi-arid and arid areas may be greater in the temperate climates we examined here than tropical and subtropical areas, because in the temperate zone soil water storage during the cool season has the potential to support a productive early growing season despite limited overall precipitation^45^. The presence of rainfed agriculture in areas previously thought to be too dry may also be the result of increasing drought tolerance of crops developed in recent decades^29^.

Our selection of important climatic variables was informed by crop yield results, but we did not directly model crop yield or the influence of enhanced atmospheric carbon dioxide concentrations on plant growth. Our projections of future suitability to support rainfed agriculture also do not incorporate the possibility of further genetic enhancements to drought resistance, or other crop specific requirements like vernalization in winter wheat. The influence of temperature extremes and soil moisture conditions over both interannual variability in crop yields and the distribution and abundance of rainfed agriculture underscores their potential utility for anticipating climate impacts on agriculture. Recent and anticipated advancements in remote sensing of cultivated areas, especially in distinguishing between rainfed vs. irrigated cultivation, will eventually lead to long-term, high spatial resolution information about cultivation trends that could test and refine our projections of future shifts in areas suitable for rainfed agriculture.

Our overall result of increasing suitability for rainfed agriculture is specific to drylands of the temperate zone. In the warmer conditions of tropical and subtropical regions, rainfed crop production is likely to be negatively impacted by rising temperatures. In fact, many of the regions identified as most at risk for declining crop yield due to climate change are in tropical and subtropical climates^6,7,35,37^, and the portions of temperate drylands that we identified as declining in suitability for rainfed agriculture tend to occur at lower latitudes. Our result of extreme high temperatures negatively affecting rainfed agricultural suitability reinforces these previous findings and illustrates how the impact of changing climate on agriculture may differ between temperate regions and tropical/subtropical regions. Although we focused only on the temperate zone, the interaction identified here between wet degree days and high temperature extremes may be useful for future studies of agricultural suitability over the entire range of drylands globally.

Cultivated agriculture is a transformative, high-impact land use practice that fundamentally alters biodiversity and ecosystem structure and function. Broad geographic changes in cultivation intensity, including both large parcels of previously cropped lands left fallow on the trailing edge as well as conversion of land to cropping due to increased suitability, would impact ecological processes and ecosystem structure at a range of spatial scales. The widespread areas of increasing suitability for rainfed agriculture in temperate drylands that we identified, combined with growing global demand for food and fiber^43^, may lead to overall agricultural intensification (especially if agricultural suitability declines in tropical and subtropical areas), and migration of cultivated land use practices into areas previously considered too cold^4^. Likewise, areas of declining suitability for rainfed agriculture, notably Europe and the southern portion of the U.S. Great Plains, may also experience substantial changes in ecosystem structure and potentially enhanced erosion and dust production as a result of crop failures. While land use decisions are influenced by many factors other than environmental suitability, changes in suitability may have dramatic impacts on ecosystems in temperate dryland regions^16^ and the non-agricultural ecosystem services that they currently provide.

## Methods

### Study sites and Regions

We defined temperate drylands as areas where a) mean annual temperature (*MAT)* > 0 °C, b) ≥ 4 but < 8 months have mean monthly temperatures ≥ 10 °C, c) Trewartha climate group category is D^46^, and d) aridity (MAP/PET, where *PET* is potential evapotranspiration and *MAP* is the mean annual precipitation) is considered arid or semiarid (0.05 ≤ AI < 0.5). We included areas meeting these temperate dryland criteria under either current conditions or future conditions for any of the scenarios we examined and our comparisons of current and future rainfed agricultural suitability utilize a consistent area (described below) which is larger than current temperate drylands. We applied a geographic raster with 0.3125-degree square cells so that exactly one cell center of the NCEP/CFSR T382 Gaussian grid^47^ fell in each of our cells (resolution of about 0.312° × ~0.312°). Our raster contained 1152 × 576 cells and had its origin at 90 °S and 179.84375 °W. From these criteria, we identified 20,020 cells for running simulations. Our results are based on 5 geographic regions from the UN geoscheme^48^: ‘South America’ (<15 °N & >25 °W); ‘Eastern Asia’ including the eastern portion of Southern Asia (along border of Afghanistan/Pakistan except area around city of Quetta) and the eastern portion of Eastern Europe (>87 °E starting about at the border point of Russia, Kazakhstan, and China); ‘Western and Central Asia’ including the western portion of Southern Asia (along border of Afghanistan/Pakistan plus area around city of Quetta) and western portion of Eastern Europe (<87 °E); ‘Western Mediterranean basin’ (W of the Dinaric and Pindus Mountains) including Europe and Northern Africa, but excluding Eastern Europe (>0 °N & (<25 °W & <14 °E); ‘North America’ (>25 °N & >50 °W).

### Data sources

As described in Schlaepfer *et al*.^49^ and Tietjen *et al*.^50^, we extracted historical (1979–2010) daily maximum and minimum temperature (2 m above ground) and 6-hourly precipitation from NCEP/CFSR data^47^. We examined two RCPs, RCP4.5 and RCP8.5, to represent emissions uncertainty, and 16 GCMs (SI Appendix, Table S3) to represent climate model uncertainty. We optimized the selection of 16 GCMs from all those that participated in CMIP5 to include the most independent and best performing subset of GCMs^51,52^ so that this optimized set of 16 GCMs likely represents more than 90% of temperature variation and more than 85% of precipitation variation among all GCMs included in CMIP5^53^. We extracted these 32 future climate conditions as monthly time-series for 2070–2099 from 1/2-degree downscaled and bias-corrected products of the fifth phase of the Climate Model Intercomparison Project (CMIP5) from the “Downscaled CMIP3 and CMIP5 Climate and Hydrology Projections” archive at http://gdo-dcp.ucllnl.org/downscaled_cmip_projections/ (data accessed on Feb 4, 2014). We combined historical daily data (NCEP/NFSR) with monthly GCM predictions of historical and future conditions with a hybrid-delta downscaling approach to obtain future daily forcing^54^.

Seasonal patterns of vegetation structure and function were estimated from climate variables as described in Bradford *et al*.^55^ and Schlaepfer *et al*.^49^ and the algorithms for estimating vegetation from climate were applied under both current climate and future climate for each time period and climate model examined. Soil texture data were derived from the ISRIC-WISE global soil dataset at 5 arc-min spatial resolution and at 20-cm depth intervals. Soil depth was estimated from ISRIC-WISE unless the soil was deeper that 1 m, in which case depth was estimated as 95% of the maximum root depth with 50-cm depth intervals^56^ and soil texture was assumed to be the same as the deepest ISRIC-WISE layer. We extracted elevation information from the GAEZ 2008 30-arcsec global elevation dataset at http://webarchive.iiasa.ac.at/Research/LUC/External-World-soil-database/HTML/global-terrain-slope-download.html?sb=7 and calculated area-weighted median and elevation range for each cell.

The proportion of each cell that is currently cultivated in dryland agriculture was estimated from the results of Teluguntla *et al*.^2^ who synthesized four previous remote-sensing studies of global croplands and developed two products relating to global cropland distribution: crop dominance and major crops, both which included estimates of rainfed vs. irrigated cultivation (report available at: http://geography.wr.usgs.gov/science/croplands/docs/Global-cropland-extent-V10-teluguntla-thenkabail-xiong.pdf) Within each simulation grid cell (0.3125° degree, about 30 km resolution) the prevalence of dryland agriculture was calculated from the remote-sensing derived agriculture estimates (1 km^2^ resolution) generated by Teluguntla *et al*.^2^. We averaged both products, with rainfed major crop abundance (Figure 6.13 in in Teluguntla *et al*.^2^) calculated as the sum of the rainfed major crop categories (classes 4 through 7: “ Rainfed: wheat, rice, and soybeans dominant”, Rainfed: wheat and barley dominant,” “Rainfed: corn and soybeans dominant,” “Rainfed mixed crops: wheat, corn, rice, barley, and soybeans”) multiplied by 0.7 to approximate the actual subpixel abundance of agriculture, based on^57^, and rainfed crop dominance (Figure 6.12c in Teluguntla *et al*.^2^) estimated as class 3 (“croplands rainfed”), class 4 “croplands, rainfed minor fragments”, and class 5 (“rainfed very minor fragments”) multiplied by 0.7, 0.25, and 0.1, respectively (reflecting within-pixel abundance^57^). Our assumptions about within-pixel abundances (i.e. the amount of a 1 km by 1km pixel identified by remote sensing as rainfed agriculture that is actually cultivated in rainfed crops) influences the regional and global estimates of land area currently cultivated. However, we explored a range of sub-pixel abundance values and found that statistical model performance and estimates of percent change in area suitable to support rainfed agriculture as a result of climate change were not substantially influenced by sub-pixel abundance values. Consequently, we focus on proportional change in suitable area by region, and present estimates of actual land area in supplementary materials. The abundance of rainfed agriculture can be influenced not only by the climatic and soil moisture conditions that support viable crop yields, but also by the economic demand for food and proximity to population centers^5^. We estimated access to markets for each cell from a dataset developed as part of the Global Land Project, accessed from (http://www.ivm.vu.nl/en/Organisation/departments/spatial-analysis-decision-support/Market_Influence_Data/index.aspx) on October 25, 2015. These market access data were highly right skewed, so we applied a natural log transformation adding a constant of 0.0001.

### Ecohydrological modeling

We utilized SOILWAT, a daily time step, multiple soil layer, process-based, simulation model of ecosystem water balance^58–60^ and specific simulations for this analysis are described in Schlaepfer *et al*.^49^. SOILWAT has been applied and found to be reliable in dryland ecosystems including temperate grasslands^58,61^ and temperate shrub-dominated ecosystems^60^. Inputs to SOILWAT include daily weather conditions (mean daily maximum and minimum temperature and daily precipitation), mean monthly climate conditions (mean monthly relative humidity, wind speed and cloud cover), latitude, elevation, vegetation (mean monthly live, standing and litter biomass, active root depth profile) and soil properties (texture of each soil layer). SOILWAT estimates processes for each functional plant group including interception by vegetation and litter, evaporation of intercepted water, transpiration and hydraulic redistribution from each soil layer. SOILWAT estimates hydrological processes including partitioning of precipitation into snowfall and rain, snow accumulation, melt and loss, infiltration into the soil profile, percolation for each soil layer, bare soil evaporation and deep drainage^59,60^. We executed these simulations on Yellowstone at the *National Center for Atmospheric Research-Wyoming Supercomputing Center* and the Advanced Research Computing Center’s Mount Moran/Bighorn facilities at the University of Wyoming.

### Statistical modeling

We considered a set of seven variables as candidates for main effects that represent specific hypotheses about environmental and economic controls over the distribution of rainfed agriculture (SI Appendix, Table S1): (1) mean annual precipitation; (2) mean annual temperature; (3) mean winter precipitation, (4) weather extremes (mean annual number of days with maximum temperature greater than 34 C; hereafter extreme hot days); (5) water availability when conditions are warm (growing degree days when soil water potential in top 20 cm was >-1.5 MPa; hereafter wet degree days); (6) mean seasonal correlation of monthly potential evapotranspiration and monthly soil water potential in shallow layers; and (7) access to economic markets (natural log transformed). These variables were selected because they are directly related to general hypotheses about the positive influence of adequate warmth and water availability as well as access to markets, and the potential negative influence of extreme high temperatures (SI Appendix, Table S1). We identified the best statistical model (as described below) for predicting rainfed agriculture from combinations of these seven main effects and first-order interactions, based on current climatic conditions and current rainfed agriculture abundance estimated from remote sensing. Each covariate was standardized prior to model fitting by centering around the mean and dividing by the standard deviation.

We modeled the number of square kilometers of dryland rainfed agriculture, *A*_*ag*_, in each cell in terms of the number of total square kilometers in the cell, *A*_*total*_ (varied from 655 to 1202 km^2^ based on latitude), the predicted proportion of land used for agricultural, *y*, (equivalent to *A*_*ag*_/*A*_*total*_) and an overdispersion term, $\theta$, to account for variation beyond that expected by a binomial response as a result of unmodelled factors. Specifically, we used beta-binomial regression where *y* was modeled as a function of covariates, *X*, and estimated coefficients, *β*, via a logit link:

$$y=\frac{\exp (\beta X)}{1\,+\,\exp (\beta X)}$$

We first selected covariates from among the seven candidates to include in our final models by a regional model selection process defined *a priori*; then, we created a set of global models using combinations of the selected covariates and interactions; finally, we ranked the global models using AIC. First, we defined three major regions (N. & S. America, East Asia, and West Asia & Europe) for model fitting. We conducted three separate model selection iterations with each major region as an out-of-sample dataset and performed forward stepwise model selection where coefficients and overdispersion parameter were estimated via maximum likelihood using the remaining two major regions as in-sample data and the performance of a model was based on the negative log-likelihood (NLL) of the out-of-sample data given the estimated *β* and $\theta$. The covariate that led to the largest drop in out-of-sample NLL was chosen at each step and forward stepwise selection continued until adding covariates no longer led to a drop in NLL. We then considered two-way interactions between covariates adding them in a stepwise fashion until addition of covariates no longer led to declining out-of-sample NLL. We repeated this process using each of the three major regions as out-of sample datasets. The best model from each region included 3 main effect predictors; 5 out of the 7 possible main effect variables were selected by at east one of the three regional optimizations. To avoid overfitting, we restricted global models to 3 out of the 5 selected main effects plus significant interactions among those main effects. We created global models that contained 3 main effects based on all combinations of the 5 candidate main effects resulting in 10 models (SI Appendix, Table S2). Coefficient estimates for each model were determined using the full dataset and estimated via maximum likelihood. Maximum likelihood estimates were reached using the optim and dbetabinom functions in R^62^ (version × 64 3.2.3), which are included in the stats^62^ and emdbook^63^ packages respectively. We used AIC to rank the 10 global models; the ranking was confirmed by RMSE (based on in-sample data) (SI Appendix, Table S2). We applied each model to estimate rainfed agriculture abundance under current climatic conditions and future climatic conditions for two representative concentration pathways and 16 general circulation models within each pathway. For each RCP, we present results from the 2^nd^, 8^th^ and 15^th^ GCM rank to represent variability among GCMs. To quantify the relative influence of statistical uncertainty vs. climate uncertainty, we partitioned variance in predictions of future land area suitable to support rainfed agriculture into variance attributable to statistical models, climate scenarios and residual variance (SI Appendix, Table S3), by dividing the sum of squares for each component by the total sum of squares for the global and regional predictions.

## Electronic supplementary material

Supplementary Material
