# Supplementary material for: Future soil moisture and temperature extremes imply expanding suitability for rainfed agriculture in temperate drylands
Source: Sci Rep. 2017 Oct 10;7:12923. doi: 10.1038/s41598-017-13165-x (PMC5635027; doi:10.1038/s41598-017-13165-x)
Supplement: Supplementary file 1 — Supplementary Material [file 41598_2017_13165_MOESM1_ESM.doc]

**Supplementary Appendix**

**Title: Future soil moisture and temperature extremes imply expanding suitability for rainfed agriculture in temperate drylands**

**Authors**: John B. Bradford^1*^, Daniel R. Schlaepfer^2,3^, William K. Lauenroth^3^, Charles B. Yackulic^1^, Michael Duniway^4^, Sonia Hall^5,6^, Gensuo Jia^7^, Khishigbayar Jamiyansharav^8^, Seth M. Munson^1^, Scott D. Wilson^9^, Britta Tietjen^10,11^

**Affiliations**

^*^Corresponding Author: [jbradford@usgs.gov](mailto:jbradford@usgs.gov); 928-523-7766

^1^U.S. Geological Survey, Southwest Biological Science Center, Flagstaff, AZ 86001, USA

^2^University of Basel, Section of Conservation Biology, 4056 Basel, Switzerland

^3^School of Forestry and Environmental Studies, Yale University, New Haven, CT 06511, USA

^4^U.S. Geological Survey, Southwest Biological Science Center, Moab, UT 86001, USA

^5^ Center for Sustaining Agriculture and Natural Resources, Washington State University, Wenatchee, Washington 98801, USA

^6^ SAH Ecologia LLC, Wenatchee, Washington 98801, USA

^7^Chinese Academy of Sciences, Institute of Atmospheric Physics, Beijing 100029, China

^8^ Department of Forest, Rangeland and Watershed Stewardship, Colorado State University, Fort Collins, CO, USA

^9^Department of Biology, University of Regina, Regina, Saskatchewan, S4S 0A2, Canada;

^10^Freie Universität Berlin, Biodiversity and Ecological Modeling, 14195 Berlin, Germany

^11^Berlin-Brandenburg Institute of Advanced Biodiversity Research (BBIB), 14195 Berlin, Germany

**Methodological Details**

We executed ecohydrological simulations on Yellowstone at the National Center for Atmospheric Research-Wyoming Supercomputing Center and the Advanced Research Computing Center’s Mount Moran/Bighorn facilities at the University of Wyoming.

***Study Site Details***: We applied a geographic raster with 0.3125-degree square cells so that exactly one cell center of the NCEP/CFSR T382 Gaussian grid(1) fell in each of our cells (resolution of about 0.312° x ~0.312°). Our raster contained 1152 x 576 cells and had its origin at 90 ˚S and 179.84375 ˚W. From these criteria, we identified 20,020 cells for running simulations. Our results are based on 5 geographic regions from the UN geoscheme(2): ‘South America’ (< 15 ˚N & > 25 ˚W); ‘Eastern Asia’ including the eastern portion of Southern Asia (along border of Afghanistan/Pakistan except area around city of Quetta) and the eastern portion of Eastern Europe (> 87 ˚E starting about at the border point of Russia, Kazakhstan, and China); ‘Western and Central Asia’ including the western portion of Southern Asia (along border of Afghanistan/Pakistan plus area around city of Quetta) and western portion of Eastern Europe (< 87 ˚E); ‘Western Mediterranean basin’ (W of the Dinaric and Pindus Mountains) including Europe and Northern Africa, but excluding Eastern Europe (> 0 ˚N & (< 25 ˚W & < 14 ˚E); ‘North America’ (> 25 ˚N & > 50 ˚W).
***Data Sources Details***: We extracted future climate conditions for the 16 GCMs and 2 RCPs as monthly time-series for 2069-2099 from 1/2-degree downscaled and bias-corrected products of the fifth phase of the Climate Model Intercomparison Project (CMIP5) from the "Downscaled CMIP3 and CMIP5 Climate and Hydrology Projections" archive at http://gdo-dcp.ucllnl.org/downscaled_cmip_projections/ (data accessed on Feb 4, 2014). We combined historical daily data (NCEP/NFSR) with monthly GCM predictions of historical and future conditions with a hybrid-delta downscaling approach to obtain future daily forcing(3). Soil depth was estimated from ISRIC-WISE unless the soil was deeper that 1 m, in which case depth was estimated as 95% of the maximum root depth with 50-cm depth intervals(4) and soil texture was assumed to be the same as the deepest ISRIC-WISE layer. We extracted elevation information from the GAEZ 2008 30-arcsec global elevation dataset at <http://webarchive.iiasa.ac.at/Research/LUC/External-World-soil-database/HTML/global-terrain-slope-download.html?sb=7> and calculated area-weighted median and elevation range for each cell. Within each simulation grid cell (0.3125˚ degree, about 30 km resolution) the prevalence of dryland agriculture was calculated from the remote-sensing derived agriculture estimates (1km^2^ resolution) generated by Teluguntla et al.(5) (report available at: <http://geography.wr.usgs.gov/science/croplands/docs/Global-cropland-extent-V10-teluguntla-thenkabail-xiong.pdf>.). We averaged both products, with rainfed major crop abundance calculated as the sum of the rainfed major crop categories (classes 4 through 7: “ Rainfed: wheat, rice, and soybeans dominant”, Rainfed: wheat and barley dominant,” “Rainfed: corn and soybeans dominant,” “Rainfed mixed crops: wheat, corn, rice, barley, and soybeans”) multiplied by a 0.7 (to approximate the actual subpixel abundance o agriculture, based on 6), and rainfed crop dominance estimated as class 3 (“croplands rainfed”), class 4 “croplands, rainfed minor fragments”, and class 5 (“rainfed very minor fragments”) multiplied by 0.7, 0.25, and 0.1, respectively (reflecting within-pixel abundance). These assumptions within-pixel abundances (i.e. the amount of a 1km by 1km pixel identified by remote sensing as rainfed agriculture that is actually cultivated in rainfed crops) influences the regional and global estimates of land area currently cultivated. However, we explored a range of sub-pixel abundance values and found that statistical model performance and estimates of percent change in area suitable to support rainfed agriculture as a result of climate change are not substantially influenced by sub-pixel abundance values. Consequently, we focus our reporting proportional change in suitable area by region, and present estimates of actual land area in supplementary materials. We represent access to economic markets using data developed as part of the Global Land Project and accessed from (<http://www.ivm.vu.nl/en/Organisation/departments/spatial-analysis-decision-support/Market_Influence_Data/index.aspx>) on October 25, 2015. These market access data were highly right skewed, so we applied a natural log transformation adding a constant of 0.0001.

**Table S1:** Independent variables considered in statistical model selection approach for predicting rainfed agriculture prevalence.

| **Variable** | **Abbreviation** | **Units** | **Hypothesized Impact** | **Justification** |
| --- | --- | --- | --- | --- |
| Mean annual temperature | MAT | C | + | Warm conditions generally support plant growth. |
| Mean annual precipitation | MAP | mm | + | Wet conditions generally support plant growth. |
| Mean winter precipitation |  | mm | + | Winter precipitation facilitates water storage during the non-growing season, potentially enabling plant growth during the warm season |
| Mean annual degree days when top 30cm of soil have soil water potential >-1.5MPa | WDD | C days | + | Warm, wet conditions in shallow soil layers should be ideal conditions for supporting cultivated agriculture. |
| Mean seasonal correlation of monthly potential evapotranspiration and monthly soil water potential in shallow layers | SEAS | unitless [-1,1] | + | Seasonal overlap between atmospheric demand (PET) and soil moisture indicates if a site's water availability patterns are coincident with the growing season |
| Mean annual days with maximum temperature > 34 C | TMAX | days | - | Extreme high temperatures can be detrimental to plant growth both by impeding cellular processes (e.g. protein denaturization) and by exacerbating drought stress |
| Access to economic markets | MKT | unitless [0,1] | + | High proximity to markets for agricultural products can maximize cultivation abundance in suitable areas and encourage cultivation in marginal locations. |

**Table S2:** Parameter estimates (and standard errors) and model fit statistics for all of the 10 global, statistical models examined. Variable abbreviations explained in Table S1.

**Table S3:** Partitioning of variance in predictions total land area suitable for rainfed agriculture into variance represented by different statistical model formulations (Table S2), alternative climate scenarios (includes all 32 combinations of representative concentration pathways and general circulation models), and residual variance.

| **Region** | **Statistical Models** | **Climate Scenarios** | **Residual** |
| --- | --- | --- | --- |
| Global | 27% | 47% | 26% |
| East Asia | 22% | 57% | 20% |
| West Asia | 24% | 47% | 29% |
| North America | 40% | 32% | 28% |
| South America | 32% | 50% | 19% |
| Europe | 25% | 31% | 44% |

**Table S4:** Global circulation models utilized, their node number in Knutti et al.’s family tree (Fig. 1B in Knutti et al.(9)) after cutting at level 16, and performance against temperature and precipitation observations, i.e., normalized distance from observations (smaller is better; Fig. 3 in Knutti et al.(9)). We utilized the highest performance GCM from each node with available information available from the "Downscaled CMIP3 and CMIP5 Climate and Hydrology Projections" archive at <http://gdo-dcp.ucllnl.org/downscaled_cmip_projections/>.

| CMIP5 GCM | Selection in our study | Node in family tree | Performance |
| --- | --- | --- | --- |
| ACCESS1.0 |  | 1 | 0.76 |
| ACCESS1.3 |  | 1 | 0.91 |
| BCC-CSM1.1 |  | 2 | 1.11 |
| BCC-CSM1.1(m) |  | NA | NA |
| BNU-ESM |  | NA | NA |
| CanESM2 | x | 10 | 0.91 |
| CCSM4 |  | 11 | 0.67 |
| CESM1(BGC) |  | 11 | 0.66 |
| CESM1(CAM5) | x | 11 | 0.60 |
| CMCC-CM |  | 4 | 0.72 |
| CNRM-CM5 |  | 2 | 0.73 |
| CSIRO-Mk3.6.0 | x | 14 | 1.19 |
| EC-EARTH | x | 2 | 0.70 |
| FGOALS-g2 | x | 15 | 0.97 |
| FGOALS-s2 | x | 12 | 0.96 |
| FIO-ESM |  | 11 | 0.98 |
| GFDL-CM3 | x | 5 | 0.76 |
| GFDL-ESM2G |  | 5 | NA |
| GFDL-ESM2M |  | NA | 0.92 |
| GISS-E2-H-CC |  | NA | NA |
| GISS-E2-R | x | 7 | 0.92 |
| GISS-E2-R-CC |  | NA | NA |
| HadCM3 |  | NA | NA |
| HadGEM2-AO |  | NA | NA |
| HadGEM2-CC | x | 1 | 0.74 |
| HadGEM2-ES | x | 9 | 0.71 |
| INM-CM4 | x | 8 | 1.32 |
| IPSL-CM5A-LR |  | 13 | 1.01 |
| IPSL-CM5A-MR | x | 13 | 0.96 |
| IPSL-CM5B-LR |  | NA | 1.28 |
| MIROC-ESM | x | 16 | 1.26 |
| MIROC-ESM-CHEM |  | 16 | 1.27 |
| MIROC4h |  | NA | 0.87 |
| MIROC5 | x | 3 | 0.78 |
| MPI-ESM-LR |  | 4 | 0.71 |
| MPI-ESM-MR | x | 4 | 0.67 |
| MRI-CGCM3 | x | 6 | 0.99 |
| NorESM1-M |  | 3 | 0.87 |
| NorESM1-ME |  | NA | 0.88 |


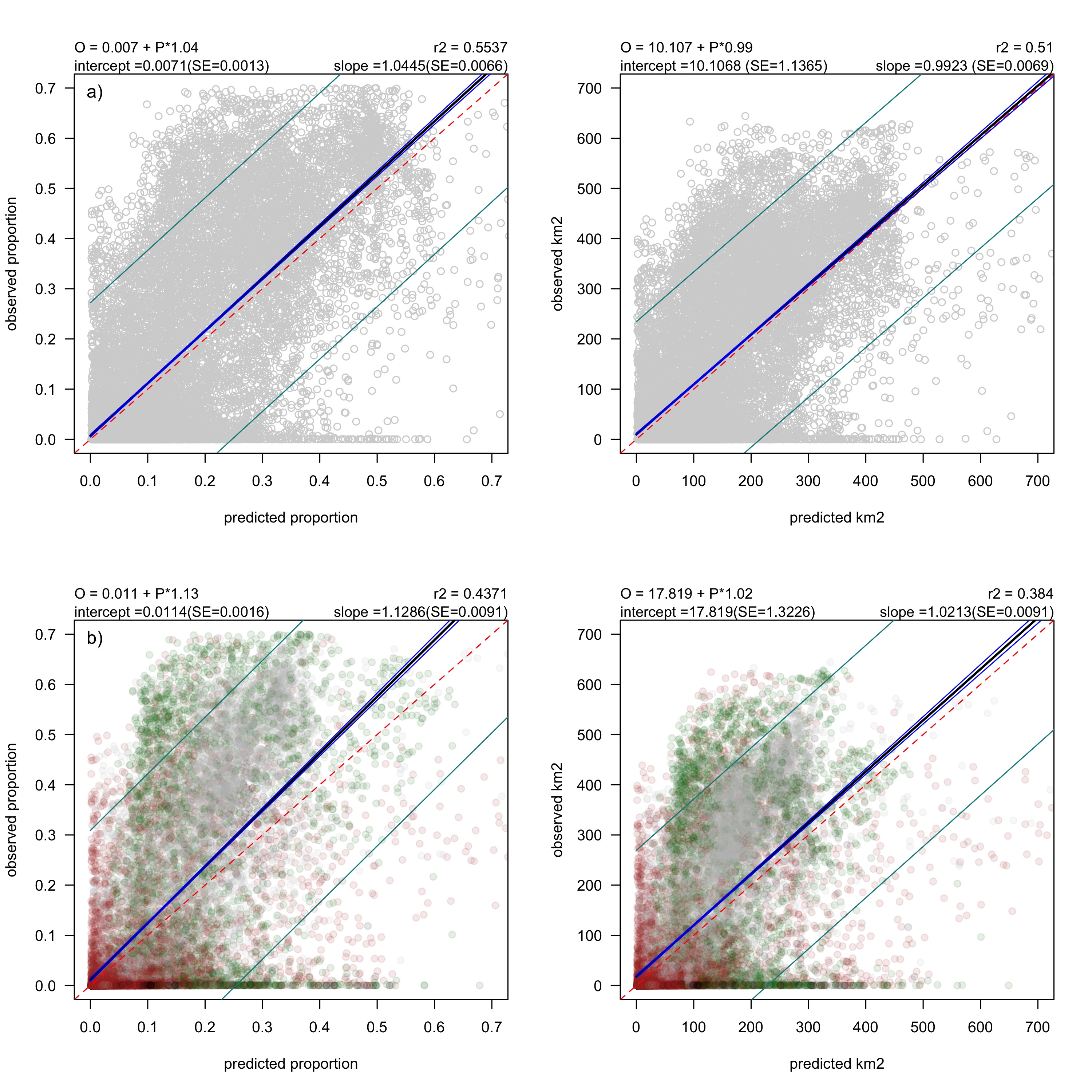
**Figure S1:** Predicted per-pixel proportion of area suitable for rainfed agriculture vs. current proportion rainfed agriculture estimated from remote sensing. Predictions are for the full model (top) and the model fitted for each region based on model fit without that region (bottom). Black line is model fit, blue lines are 95% confidence intervals, cyan lines are 95% prediction intervals and dotted red line is 1:1. Point colors in bottom show major region: red=East Asia, gray=West Asia & Europe, green=North & South America.

**Figure S2:** Maps of mean annual precipitation, mean annual temperature and market access (1=high access). Maps for wet degree days and temperature extremes are presented in Figure S6 and S7, respectively. Created in R version3.3.2.


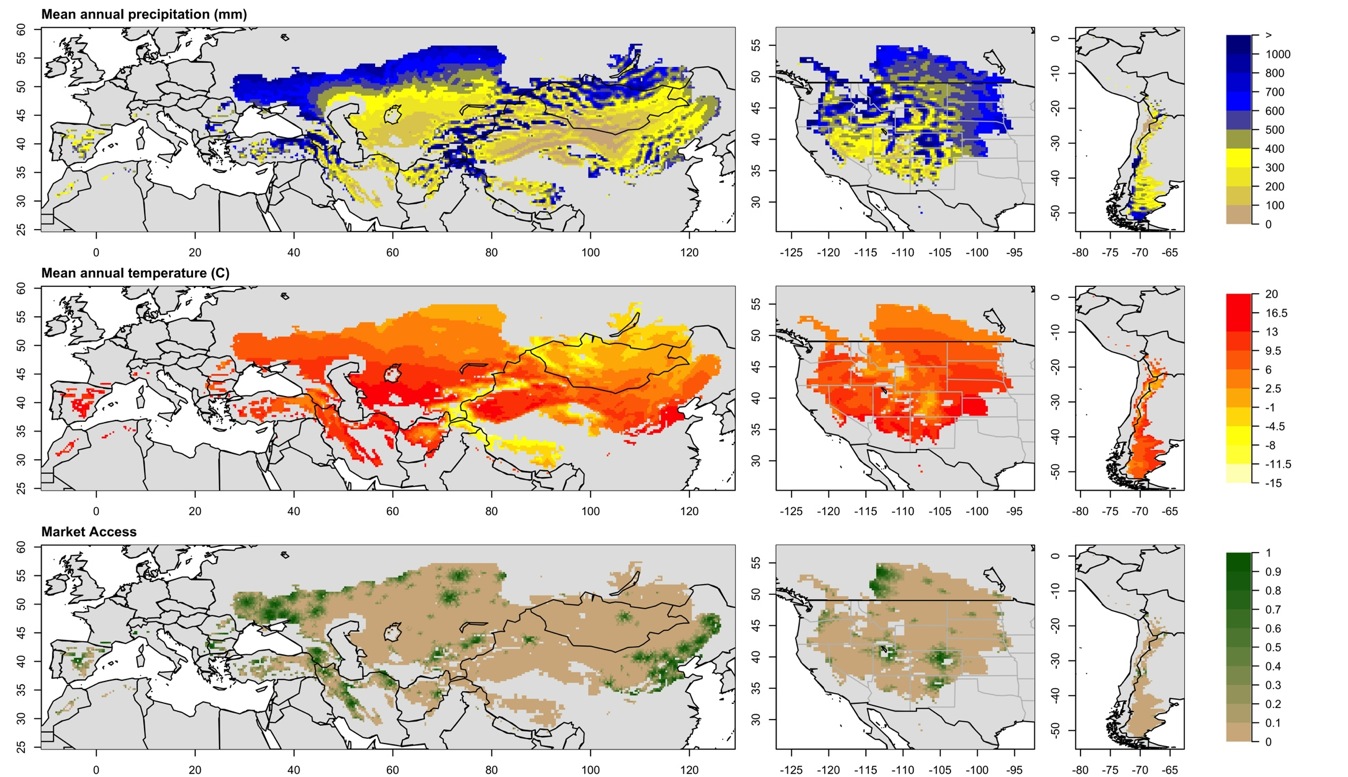


**Figure S3:** Difference in proportion dryland agriculture between the final model and estimated from remote sensing. Blue indicates where modeled predictions are higher than remote sensing, and orange area areas where they are less. Created in R version 3.3.2. ([www.Rproject.org/)](http://www.Rproject.org/)).
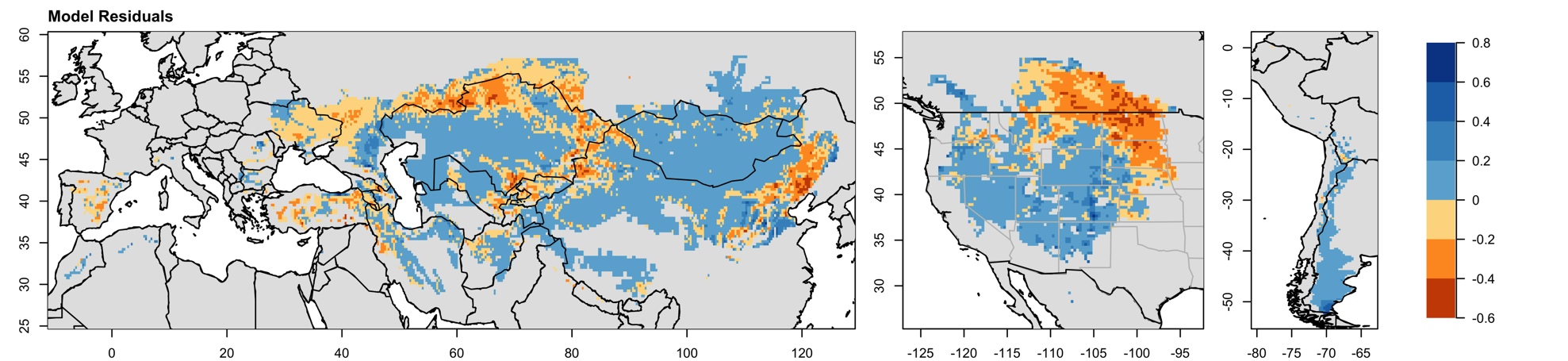


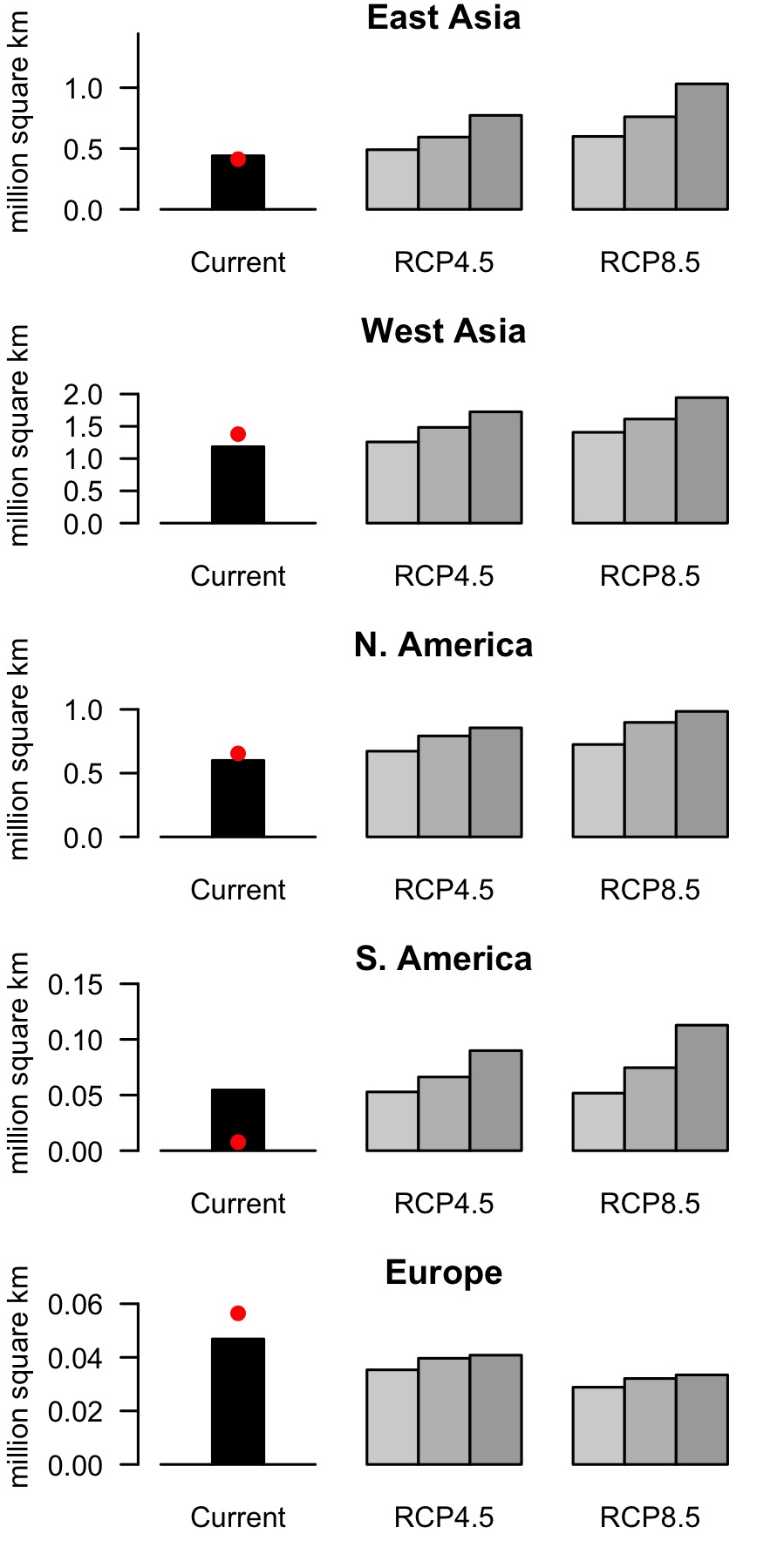
**Figure S4:** Estimated land area suitable for rainfed agriculture under current climatic conditions (black bar), and future climatic conditions for RCP 4.5 (center group) and RCP8.5 (right group), assuming within-pixel abundances as described in the text. Red dot indicates current estimate of rainfed agriculture observed from remote sensing. Estimates for three GCMs (rank 2, 8 and 15 out of 16 GCMs examined) are shown for each RCP in light, medium and dark gray bars, respectively. Note the different scales in differing regions.

**Figure S5:** Rainfed agriculture (expressed as proportion of land area) in temperate drylands estimated from: A) remote sensing (e.g. realized abundance), B) predicted under current conditions from a statistical model (e.g. potential abundance), C) predicted under future conditions (median GCM, RCP4.5), and D) change in prevalence between future and current climate. Created in R version 3.3.2. ([www.R-project.org/)](http://www.R-project.org/)).
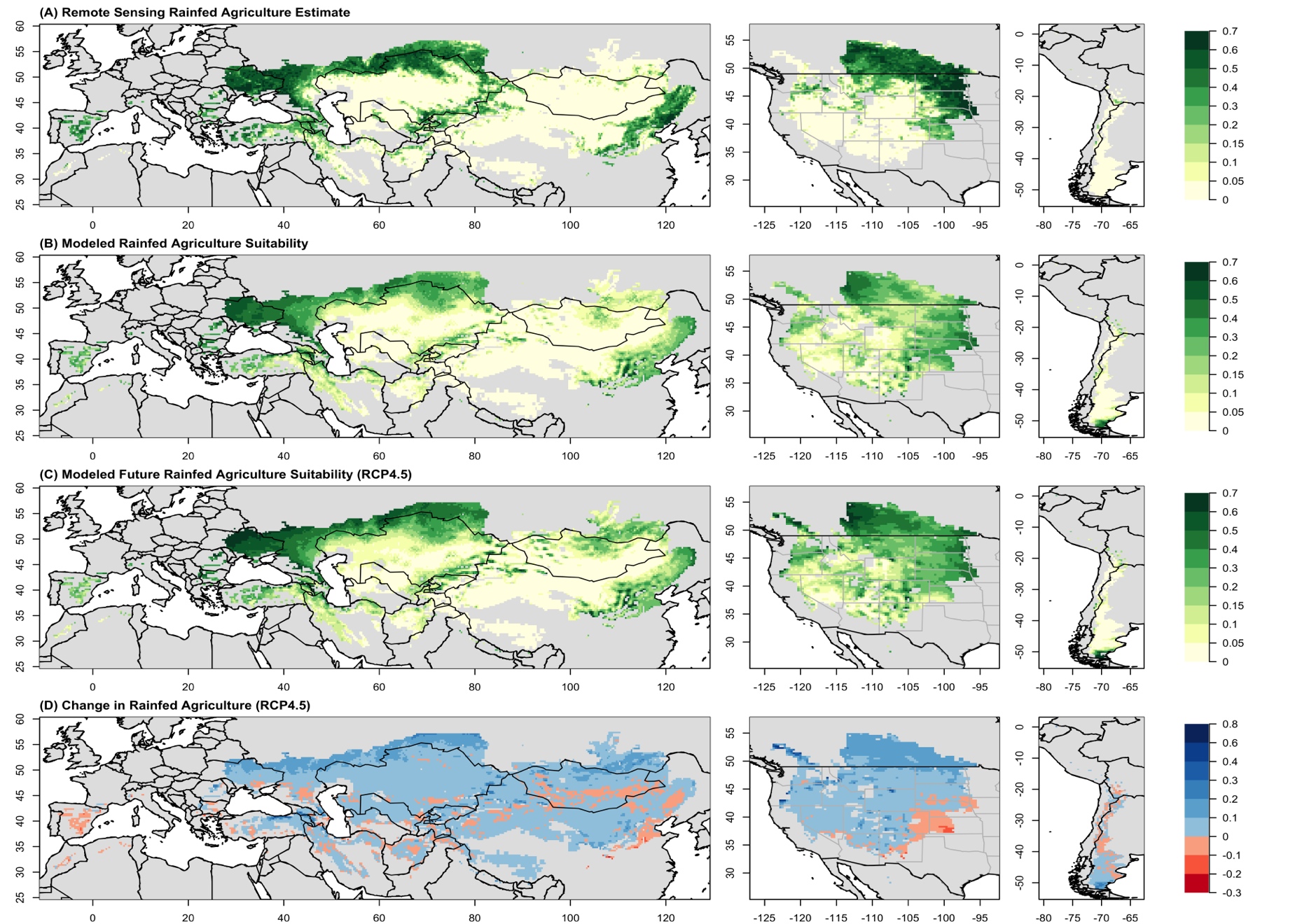


**Figure S6:** Wet degree days in the top soil layers (WDD) and change in WDD between current and future conditions (median GCM, RCP4.5 and RCP8.5) for temperate dryland regions. Created in R version 3.3.2. ([www.R-project.org/)](http://www.R-project.org/)).


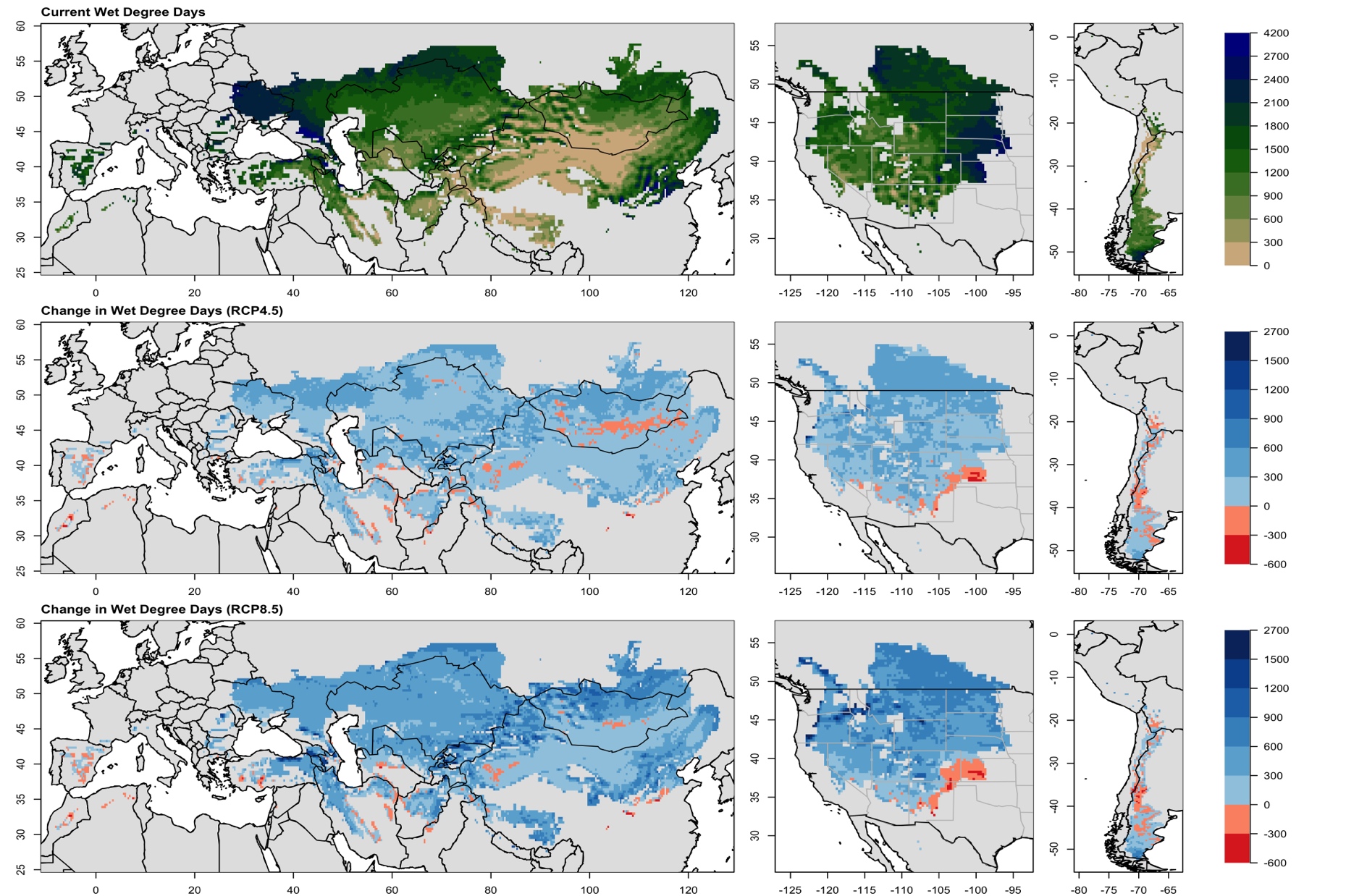


**Figure S7:** Number of days with maximum temperature greater than 34**°**C (TMAX), and change in TMAX between current and future conditions (median GCM, RCP4.5 and RCP 8.5) for temperate dryland regions. Created in R version 3.3.2. ([www.R-project.org/)](http://www.R-project.org/)).


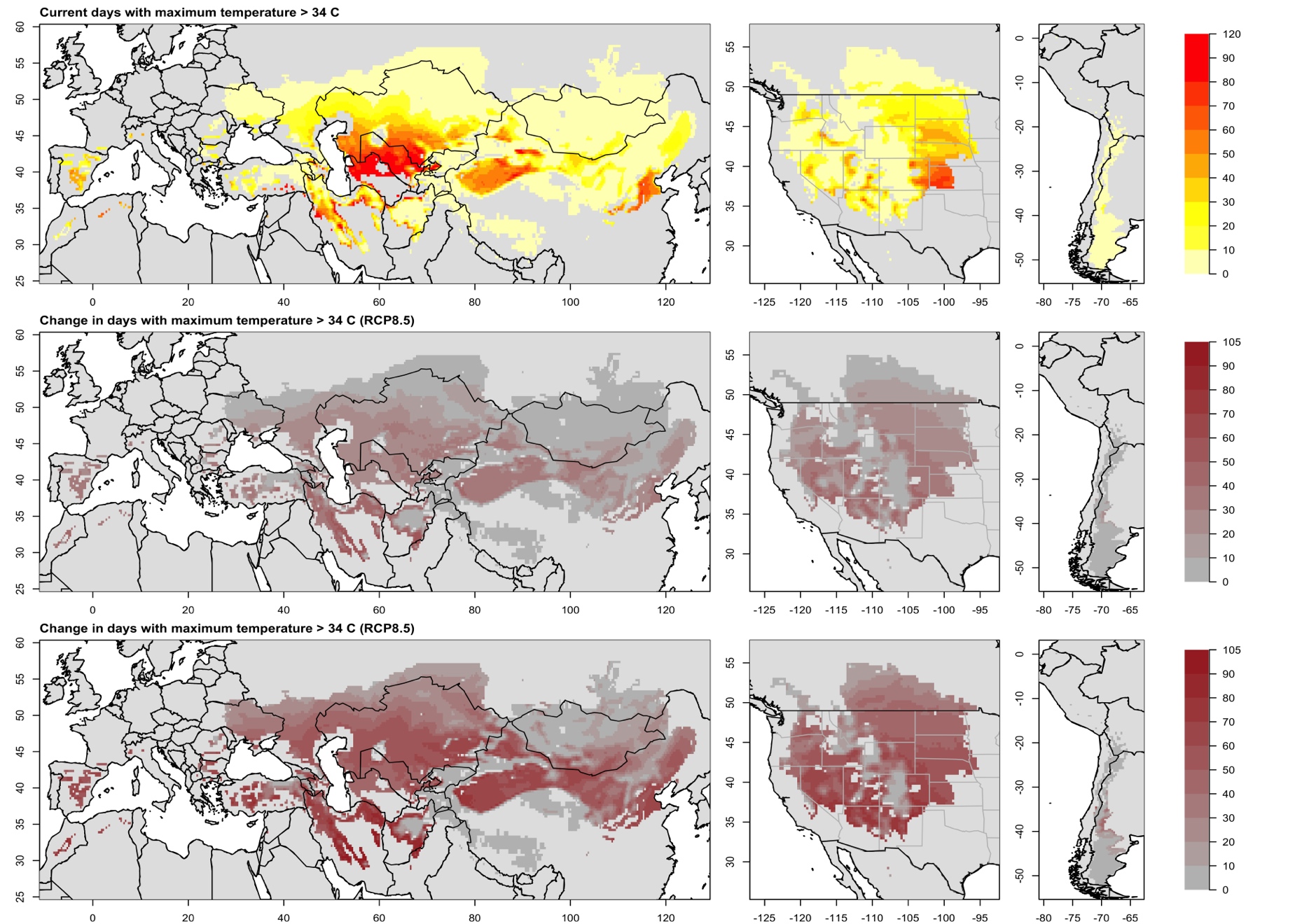


**Figure S8:** Sensitivity of changes in area suitable for rainfed agrticulture to changes in mean annual precipitation (left) and temperature (right) for each region examined. Lines depict variability among GCMs (using minimum, median and maximum) under RCP 4.5 (blue) and RCP 8.5 (red). Created in R version 3.3.2. ([www.R-project.org/)](http://www.R-project.org/)).


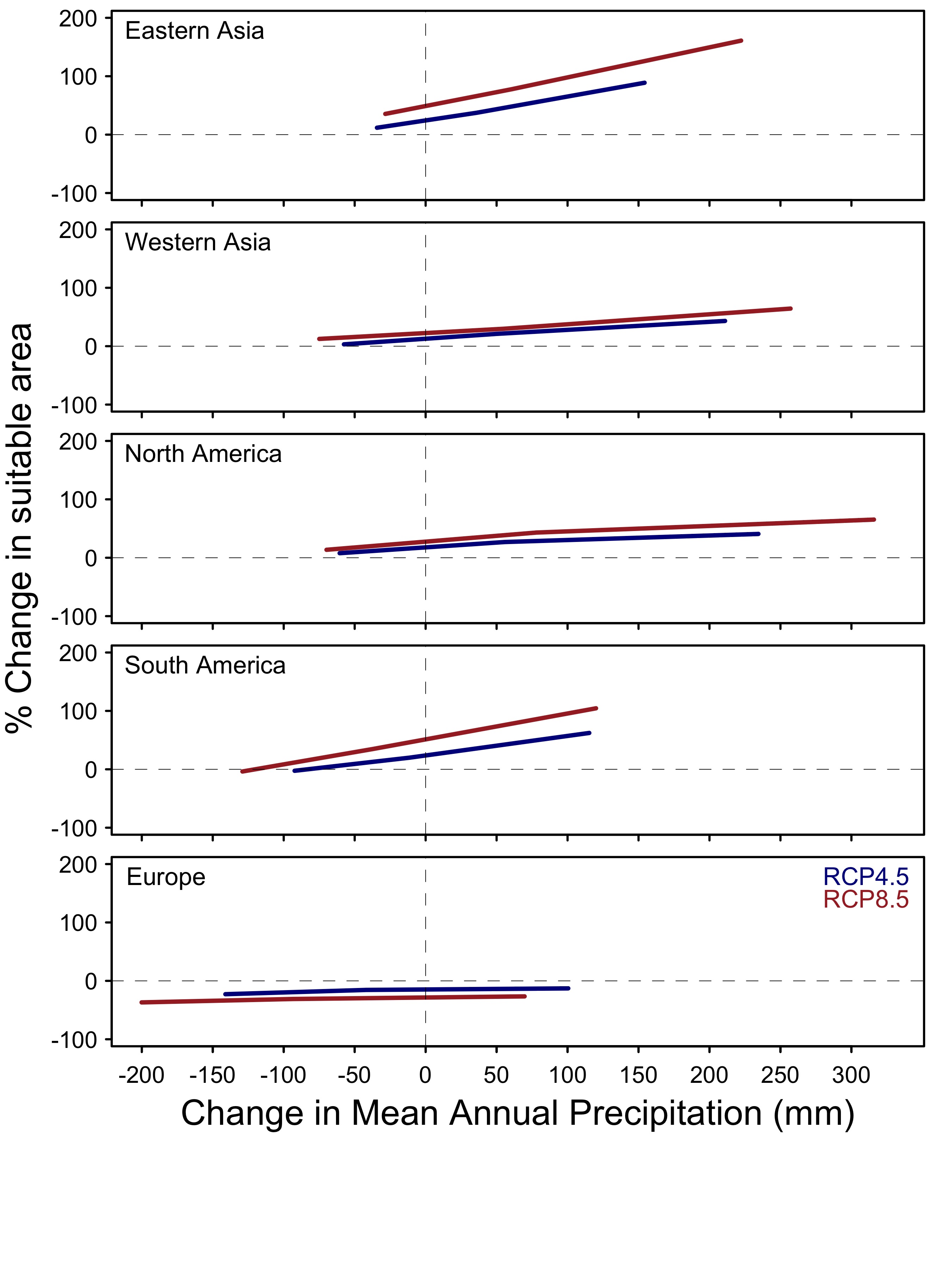

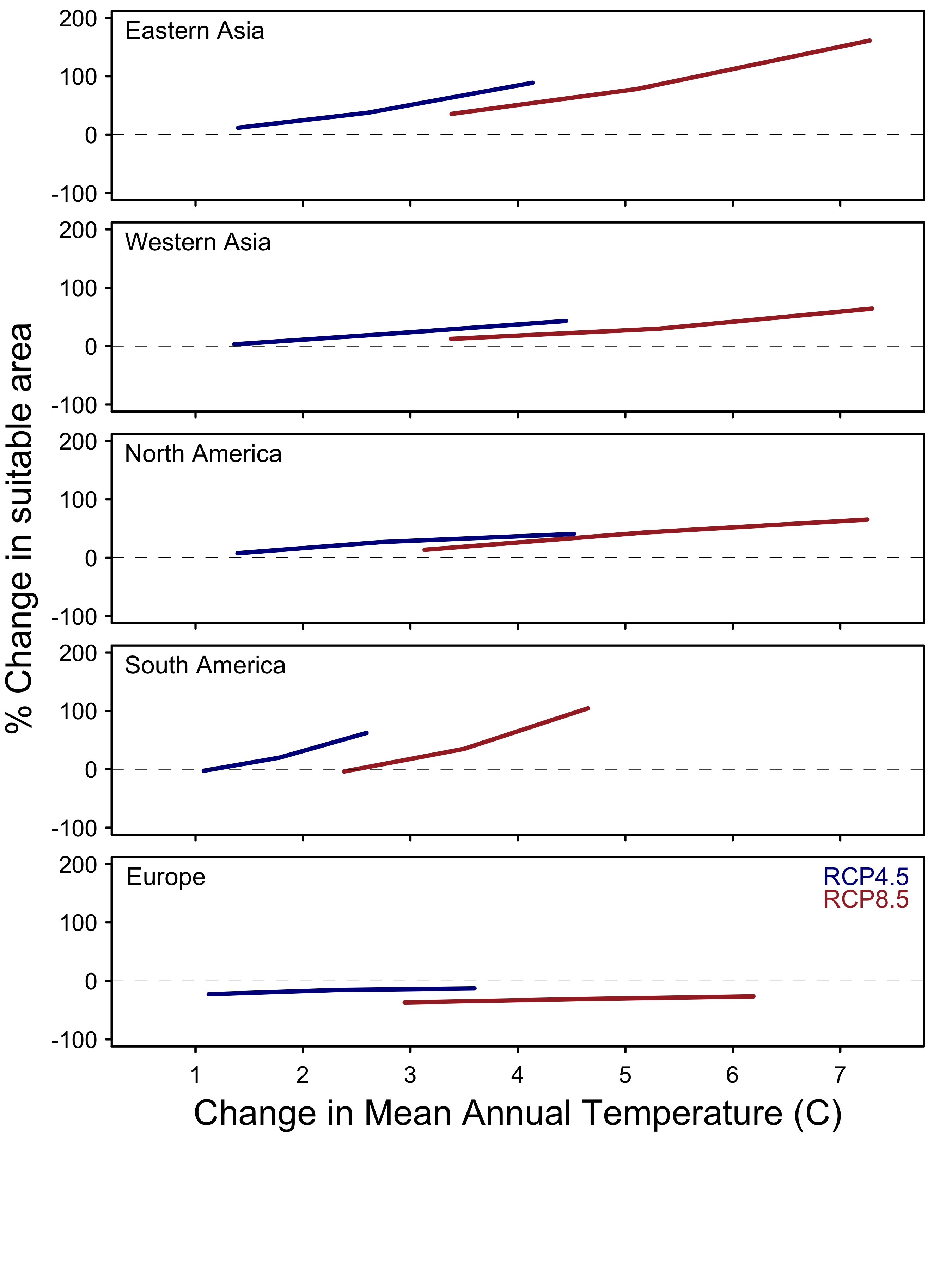


REFERENCES
